# Assessment of Anatomical Variations in the Sacroiliac Joint Using Magnetic Resonance Imaging: A Retrospective Study of 840 Patients

**DOI:** 10.3390/diagnostics16071020

**Published:** 2026-03-28

**Authors:** Selen Beyazıt, Gezmiş Kimyon, Sinem Karazincir

**Affiliations:** 1 Department of Radiology, Arsuz State Hospital, 31285 Hatay, Turkey; 2 Department of Rheumatology, Mustafa Kemal University, 31040 Hatay, Turkey; gkimyon@gmail.com; 3 Department of Radiology, Mustafa Kemal University, 31040 Hatay, Turkey; sinemkarazincir@yahoo.com

**Keywords:** sacroiliitis, magnetic resonance imaging, sacroiliac joint variations, bone marrow edema

## Abstract

**Background/Objectives:** This study aimed to examine the prevalence of anatomical variations in the sacroiliac joints (SIJs) as observed through Magnetic Resonance Imaging (MRI), to characterize their manifestations, and to identify MRI features that may resemble inflammatory alterations. **Methods:** A retrospective review was conducted on consecutive MRI scans of the SIJ performed from January 2009 to January 2022. Eight anatomical variations, along with associated edematous and structural changes, were assessed. **Results:** The study encompassed 840 patients, with anatomical variations identified in 39.7% of the cohort, occurring more frequently among female participants. The most prevalent variations were accessory SIJ (36.2%) and the iliosacral complex (32.2%). Notably, isolated synostosis and persistent ossification center variations were absent. The increased frequency of variations in women, as well as their correlation with advancing age, was statistically significant (*p* = 0.034). Accessory SIJ and dysmorphic alterations were linked to bone marrow edema and structural modifications. In the iliosacral complex and semicircular defect variations, prominent vascular structures were observed extending along the bone surfaces. The number and depth of edema slices in sacroiliitis exceeded those observed in the variation (*p* < 0.001). **Conclusions:** Anatomical variations of the SIJ are prevalent among women and tend to increase with advancing age. Given that these variations, particularly accessory SIJ and dysmorphic alterations, may present with edematous and structural signal intensity changes that resemble sacroiliitis, it is crucial to recognize these variations. It is recommended to assess axial and coronal images concurrently and to exercise caution in the interpretation of SIJ MR images.

## 1. Introduction

Imaging techniques of the sacroiliac joints (SIJs) are integral to the diagnostic process for axial spondyloarthritis (ax SpA), as they facilitate the identification of structural and inflammatory lesions indicative of sacroiliitis. Magnetic Resonance Imaging (MRI) is regarded as the most sensitive modality for detecting early inflammatory changes in the SIJ associated with arthropathy.

MRI findings such as subchondral edema, synovitis, enthesitis, and capsulitis, which indicate active sacroiliitis, as well as erosions, subchondral sclerosis, periarticular fat deposition, and ankylosis, which suggest chronic sacroiliitis, are delineated in the diagnostic criteria established by the Assessment of SpondyloArthritis International Society (ASAS) [1].

Bone marrow edema in the SIJ constitutes the primary imaging characteristic of ax SpA. Bone marrow edema that satisfies the ASAS criteria has been documented in anatomical variations of the SIJ. Furthermore, structural alterations such as fatty infiltration and/or sclerosis have been identified in certain variants, complicating the MRI assessment of the SIJ. In evaluating the SIJ, it is crucial to recognize the variations present in the joint, the associated edematous changes in the adjacent bone marrow, and the structural signal intensity changes (including subchondral sclerosis, subchondral cysts, osteophytes, and fat deposition). Additionally, assessing the localization and extent of the observed edema is essential to prevent diagnostic errors.

The SIJ can present with various morphological variations and anatomical localizations [2]. In the literature, studies examining the prevalence of these variations predominantly utilize computed tomography (CT), with reported prevalence rates ranging from 36.3% to 52.4% [2,3,4,5]. However, research employing MRI remains limited, and a comprehensive characterization of MRI findings for these variations has yet to be fully established [6,7]. This gap in the literature may lead to diagnostic challenges and potential false-positive diagnoses of sacroiliitis in clinical practice.

The aim of this study is to investigate the frequency of SIJ anatomical variations using MRI, to delineate the detailed MRI characteristics of each variation type, and to highlight the MRI features of those variations that may pose diagnostic difficulties.

## 2. Materials and Methods

### 2.1. Patient Selection and Study Design

A retrospective analysis was conducted on the medical records of 856 consecutive patients who underwent SIJ MRI between January 2009 and January 2022. The MRI examinations were performed based on clinical indications as determined by the referring physicians. The most prevalent indications included inflammatory low back pain, suspected sacroiliitis, evaluation of spondyloarthritis, chronic low back pain, and differential diagnosis of sacroiliac joint pathologies.

This retrospective study received approval from the Hatay Mustafa Kemal University Ethics Committee on 13 January 2022 (decision number: 2022-06). Given the retrospective nature of the study and the utilization of anonymized data, the requirement for informed consent was waived. The study adhered to the principles outlined in the Declaration of Helsinki. Patients with inadequate image quality due to motion artifacts or technical issues, as well as those with tumors, pseudotumors, fractures, septic arthritis, or a history of sacroiliac joint surgery, were excluded. After applying these exclusion criteria, a total of 840 patients were included in the final analysis.

Demographic and clinical data, including age, sex, presenting complaints, laboratory findings, and clinical diagnoses, were retrospectively obtained from patient records and electronic databases.

While the presenting complaints, laboratory findings, and clinical diagnoses were examined, laboratory parameters pertinent to axial spondyloarthritis—such as HLA-B27 status, C-reactive protein (CRP), and erythrocyte sedimentation rate (ESR)—were not consistently available for all patients and, consequently, were excluded from the statistical analysis.

**Figure 1:**
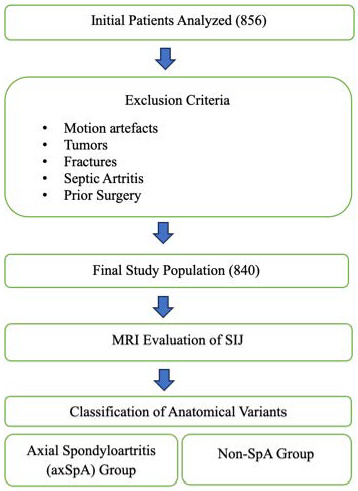
Patient Selection of Study.

The categorization of patients into the axSpA cohort was predicated on the diagnosis recorded by the referring clinician in the medical documentation. Patients were stratified into two cohorts based on their clinical diagnoses: axSpA and non-spondyloarthritis (non-SpA). The axSpA cohort comprised individuals diagnosed with axial spondyloarthritis by a clinician. Conversely, the non-SpA cohort included patients who underwent SIJ MRI for various clinical indications but exhibited no clinical or laboratory evidence of spondyloarthritis and did not satisfy the ASAS classification criteria for axSpA. This group predominantly consisted of patients assessed for chronic low back pain or other non-inflammatory conditions (Figure 1).

### 2.2. MRI Review Protocol

The MRI examination was conducted utilizing both a 1.5-Tesla MR system (Philips Ingenia, Best, The Netherlands) and a 3-Tesla MR system (GE SIGNA Architect, Waukesha, WI, USA), employing a spinal coil with the patient in the supine position. The standard imaging protocol comprised paracoronal T1-weighted (T1W), paracoronal short tau inversion recovery (STIR), and paraxial fat-suppressed T2-weighted (T2W) sequences. All images were acquired with a slice thickness of 3 mm, with the paraxial and paracoronal planes oriented parallel and perpendicular, respectively, to the superior plateau of the first sacral vertebra.

### 2.3. MRI Analysis and Evaluation of Anatomical Variations

All SIJ MRI examinations were independently assessed by two radiologists with 24 and 5 years of experience in musculoskeletal imaging, respectively. Both evaluators were blinded to the patients’ clinical data and diagnoses during the image interpretation process. Following the independent assessments, all images were jointly reviewed, and a consensus was reached in all instances. Findings agreed upon by both radiologists were accepted as present. In cases of initial disagreement, the final decision was established through consensus. Interobserver agreement was not calculated, as all evaluations were resolved by consensus.

**Figure 2:**
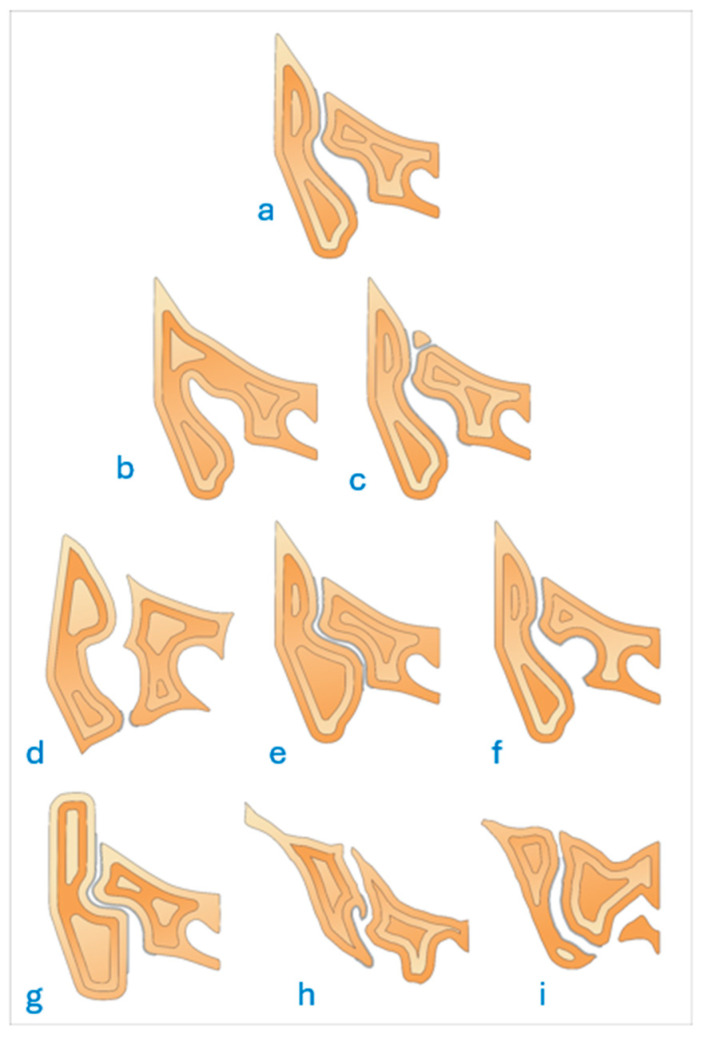
Schematic drawing of the eight anatomical variations. (**a**) normal sacroiliac joint, (**b**) isolated synostosis, (**c**) persistent ossification center, (**d**) accessory sacroiliac joint, (**e**) iliosacral complex, (**f**) semicircular defect, (**g**) dysmorphic change, (**h**) bipartite iliac bone plate, and (**i**) crescent ilium.

A total of eight anatomical variations observed in the SIJ were evaluated as defined below [2,6] (Figure 2).

Accessory SIJ: A joint formed between the sacral and iliac bones posterior to the cartilaginous part of the joint.

Iliosacral Complex: A protrusion extending from the iliac bone toward the sacral bone, with a corresponding indentation in the sacral bone.

Bipartite Iliac Bone Plate: A double-layered appearance of the iliac bone.

Semicircular Defect: A defect-like, half-moon appearance on the iliac and sacral surfaces.

Crescent Ilium: The part of the iliac bone facing the sacroiliac surface is crescent-shaped, accompanied by a protrusion on the sacral surface.

Persistent Ossification Center: A triangular-shaped ossification center belonging to the sacral ala, located anterior to the SIJ.

Isolated Synostosis: Presence of focal ankylosis without structural or inflammatory changes.

Dysmorphic Change: An elongated posterior joint appearance with a convex sacral surface border protruding into the iliac bone.

In patients where anatomical variations were detected, the optimal imaging plane (coronal, axial, or both) for visualizing the variation, laterality (unilateral or bilateral), involvement of the sacral foramina, any accompanying edematous changes (at least two consecutive slices), and/or structural signal intensity changes (subchondral sclerosis, subchondral cysts, osteophytes, and fat accumulation), as well as the presence of prominent vascular structures near these variations, were assessed.

The quantification of slices and the depth of edema in each SIJ, alongside the presence of subchondral hyperintensity bone marrow edema in at least two regions and/or on at least two consecutive slices in the STIR and para-axial fat-suppressed T2W sequences, was assessed as bone marrow edema.

The anatomical localization of the variation was delineated, as described in the study by Giruardo et al., on paracoronal and para-axial images: the upper part of the first sacral foramen was designated as the superior one-third, the area between the first and second sacral foramina as the middle one-third, and the distal part of the second sacral foramen as the inferior one-third. On axial images, the demarcation between the anterior and posterior parts of the joint was established at the transition points between the cartilaginous and ligamentous sections [8].

### 2.4. Statistical Analysis

The research data were analyzed utilizing the SPSS 22.0 statistical software package. The normality of the distribution was assessed both visually, through histograms and probability plots, and analytically, using the Kolmogorov–Smirnov and Shapiro–Wilks tests. Descriptive statistics for parametric data were expressed as mean and standard deviation, whereas non-parametric data were represented by median and minimum–maximum values. For statistical methods, the Chi-square test was employed for categorical variables, the Mann–Whitney U test was applied to determine statistical significance between two independent groups, and the Kruskal–Wallis test was used for assessing statistical significance among more than two independent groups. A *p*-value of less than 0.05 was considered indicative of statistical significance.

## 3. Results

Of the 840 patients who participated in the study, 324 were male (38.6%) and 516 were female (61.4%). The mean age was 38.7 ± 12.2 years, with a range of 18 to 79 years. Low back pain was the presenting complaint in 639 patients (76.1%). A total of 137 patients (16.3%) were classified within the axSpA group, while 703 patients (83.7%) were categorized in the non-SpA group.

Among the 840 patients whose SIJ MRI was evaluated, at least one anatomical variation was identified in 334 patients (39.7%). The study identified two different variations in 20 patients (2.38%), resulting in a total of 354 variations being assessed. The most frequently co-occurring variations were the accessory SIJ and the iliosacral complex, observed in 4 patients.

Upon evaluating anatomical variations according to their frequency, calculated over the total number of variations (*n* = 354), the distribution was observed as follows: 1. Accessory SIJ (128 patients, 36.2%), 2. Iliosacral complex (114 patients, 32.2%), 3. Semicircular defect (44 patients, 12.4%), 4. Crescent ilium (26 patients, 7.3%), 5. Bipartite iliac bone plate (24 patients, 6.8%), and 6. Dysmorphic changes (18 patients, 5.1%). The frequency of SIJ anatomical variations is summarized in Table 1. In this study, neither persistent ossification center nor isolated synostosis variations were observed.

In this study, SIJ variations were identified bilaterally in 194 patients (58.1%) and unilaterally in 140 patients (41.9%). Among those with variations, 260 (77.8%) were female and 74 (22.2%) were male, with a significantly higher prevalence in females (*p* < 0.001). The incidence of variations was positively correlated with increasing age (*p* < 0.001).

**Figure 3:**
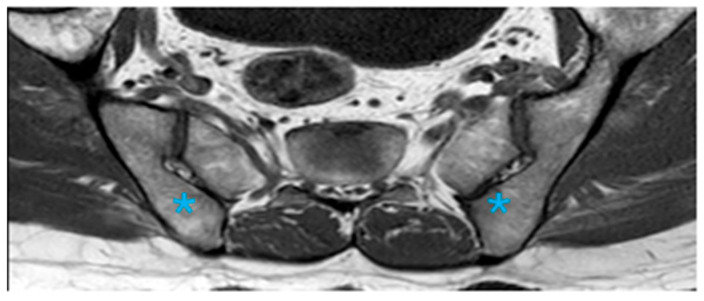
The accessory SIJ is formed between the sacral and iliac bones in the posterior middle third of the paraxial T1A and the joint (Asterisk; bilateral accesory SIJ).

An accessory SIJ was the most prevalent variation, detected in 128 patients (36.2%), and was more frequently observed in females (56.9%). This variation was optimally visualized on axial images, with unilateral presentation in 79 patients (61.7%) and bilateral presentation in 49 patients (38.3%). The accessory SIJ was predominantly located between the first and second sacral foramina, in the middle third of the posterior region. Edematous changes in the adjacent bone marrow were noted in 6 patients (4.7%), while structural signal intensity alterations were observed in 60 patients (46.9%). Accompanying structural changes included sclerosis, fatty replacement, joint space narrowing, and ankylosis, with sclerosis being the most frequently observed alteration (Figure 3).

**Figure 4:**
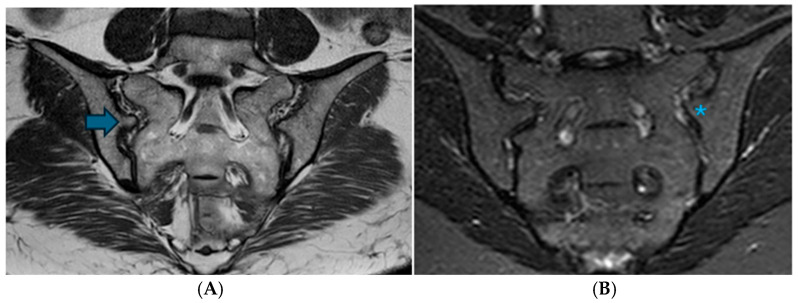
The bilateral iliosacral complex is depicted in paracoronal T1W images in (**A**) and paracoronal STIR images in (**B**). There is a protrusion extending from the iliac bone to the sacral bone at the posterior superior one-third of the joint, accompanied by a corresponding indentation in the sacral bone (Arrow: iliosacral complex; asterisk: prominent vascular structures).

The iliosacral complex was identified in 114 patients (32.2%), representing the second most prevalent variation, with a higher incidence in women (87.2%). Optimal visualization was achieved in the coronal plane, with bilateral detection in 74 patients (64.9%) and unilateral detection in 40 patients (35.1%). This complex was predominantly observed at the level of the first sacral foramen, specifically in the superior posterior one-third of the joint. Sclerosis was noted on the bone surfaces adjacent to the variation in 6.1% of the patients, while prominent vascular structures were associated with the variation in 42.1% of the cases (Figure 4).

In a cohort of 44 patients (12.4%), a semicircular defect was identified, ranking as the third most prevalent variation. This defect was predominantly observed in female patients (91.9%). Optimal detection occurred in the coronal plane, with the defect most frequently located above the first sacral foramen, specifically in the superior posterior one-third of the joint, and it was often bilateral (81.8%). Notably, in 52.3% of cases exhibiting the semicircular defect, prominent vascular structures were observed extending along the defect [6].

**Figure 5:**
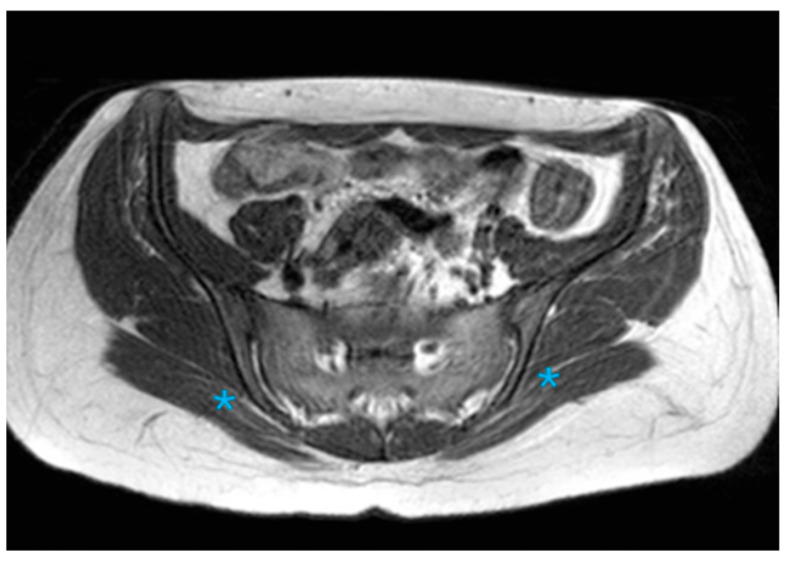
Crescent ilium, paraxial T1A image. The iliac bone exhibits a crescent-shaped morphology on the portion facing the sacroiliac surface, specifically in the middle third of the posterior joint. This is accompanied by a protrusion on the sacral surface (Asterisk; bilateral crescent ilium).

A crescent ilium was identified in 26 patients (7.3%), all of whom were female. This variation was predominantly located between the first and second sacral foramen, specifically in the middle third of the posterior region. It was most effectively detected on the axial plane and was frequently bilateral, occurring in 92.3% of cases (Figure 5).

A bipartite complex was identified in 24 patients (6.8%), all of whom were female, particularly those presenting with a crescent ilium. This anomaly was most frequently located between the first and second sacral foramina, specifically in the middle third of the posterior section. The variation was predominantly bilateral (79.2%) and was most effectively visualized on the axial plane. Additionally, a canal-like formation was observed at this level in 58.3% of the patients.

**Figure 6:**
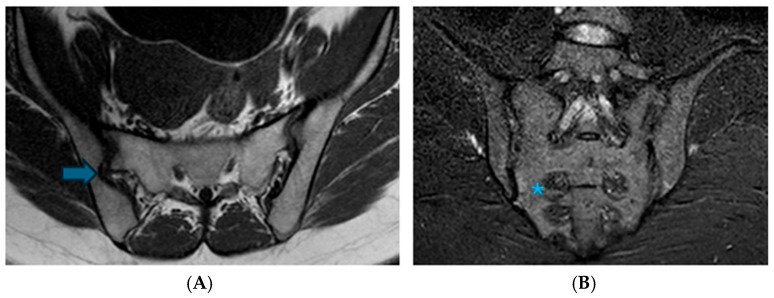
Illustrates dysmorphic changes. Image (**A**) presents paraxial T1A, while image (**B**) displays paracoronal STIR images. In the distal one-third anterior section of the joint, the cartilage cut reveals a convex sacral surface border extending towards the iliac bone (Arrow: dysmorphic changes; asterisk: edematous changes in the adjacent sacral bone).

Dysmorphic changes were identified in 18 patients (5.1%), representing the least frequently detected variation. The majority of these patients were female (77.8%). The variation was predominantly located beneath the second sacral foramen, anterior to the inferior third of the joint. It was primarily unilateral (94.4%) and was most effectively detected on axial images. Accompanying structural signal intensity changes were observed in 44.4% of patients as edema, and in 55.6% as sclerosis and subchondral cysts (Figure 6).

Table 2 provides a summary of the frequency, laterality, location, and associated osseous changes of SIJ anatomical variations.

In patients exhibiting identified variations, bone marrow edema was detected at the site of the variation in the sacrum and ilium. The average number of slices displaying edema associated with these variations was 2.5 ± 0.6, with a mean edema depth of 6.1 ± 2.4 mm. In the axSpA group presenting with sacroiliitis, edema was predominantly observed bilaterally, occurring in both the sacrum and ilium in 68.3% of cases. Regarding localization, edema was most frequently identified in the superior one-third locality and the anterior part of the joint, specifically in the cartilage section. The mean number of slices with edema observed in sacroiliitis was 6.4 ± 3.5, and the mean edema depth was 10.5 ± 3.5 mm. Both the number of slices and the depth of edema observed in sacroiliitis were statistically significantly greater than those associated with variations (*p* < 0.001).

## 4. Discussion

This study constitutes one of the most extensive patient series in which anatomical variations of the SIJ are assessed using MRI. The objective of the research was to examine the prevalence of these anatomical variations, to delineate the detailed MRI characteristics of each variation type, and to emphasize the MRI features of those that may present diagnostic challenges.

In our study, a variation was identified in 39.7% of cases, occurring more frequently among women. This finding aligns with previous research utilizing CT and/or MRI, which reported a variation rate of 32–55% [2,3,4,5,6,7]

The accessory SIJ and the iliosacral complex are the most commonly observed anatomical variations, with prevalence rates of 36.2% and 32.2%, respectively. These findings are consistent with the results reported by Demir [4] and Prassopoulos [2].

The accessory SIJ was most clearly visualized in the axial plane at the middle third of the posterior section, whereas the iliosacral complex was observed in the coronal plane and within the superior third of the posterior section of the joint. Our study did not identify any instances of isolated synostosis or variations in a persistent ossification center.

Variations identified in different studies exhibit considerable diversity. In the investigations conducted by Rafei [6] and Kiil [7], the most prevalent dysmorphic change was observed, whereas Cihan [3] and Tok [9] identified the iliosacral complex as the most common. It is posited that these discrepancies in prevalence may be attributed, in part, to differences in study populations, the imaging modalities employed, technical variations in imaging, discrepancies in the definition of anomalies, and the absence of definitive size criteria.

In anatomical variations of the SIJ, bone marrow edema that meets the ASAS criteria has been documented in healthy individuals, athletes, and those experiencing mechanical low back pain [6,7,10,11,12]. Variations in the cartilaginous and ligamentous components of the SIJ, along with associated edematous and structural signal intensity changes, have been occasionally reported in a limited number of studies to mimic certain pathologies—particularly sacroiliitis—thereby posing diagnostic challenges [6,7]. Consequently, the location, extent, and intensity of bone marrow edema, as well as the presence of specific structural lesions such as erosion, are critical for differential diagnosis. In athletes and healthy individuals, the edema is typically of low grade, predominantly affecting the posterior inferior ilium, followed by the anterior superior sacrum [11,13]. Edema and sclerosis resulting from mechanical changes are generally observed in the mid-anterior sacrum, limited to no more than four consecutive slices, confined to the specified localization, small in size, and without significant erosion [12]. Conversely, edema associated with sacroiliitis is reported to be more extensive and intense, accompanied by structural changes in the ilium and sacrum that also involve the ligamentous compartments [10,12,14]. In our study, edema in patients with sacroiliitis was most frequently bilateral and more commonly observed in the cartilage portion at the anterior superior site, with the number and depth of edema slices exceeding those observed in variations.

The lack of comprehensive clinical and laboratory data may further complicate the differentiation between anatomical variation-related MRI findings and true inflammatory sacroiliitis in routine clinical practice. Specifically, parameters such as HLA-B27 status and inflammatory markers (CRP, ESR), which support the diagnosis of axial spondyloarthritis, were not consistently available in this study. In daily practice, these clinical and laboratory findings are typically interpreted alongside MRI to enhance diagnostic confidence. Their absence may increase the risk of misinterpreting mechanically induced or variation-related bone marrow edema and structural changes as inflammatory sacroiliitis. Therefore, careful assessment of the location, extent, and pattern of MRI findings becomes even more critical in such contexts. Despite this limitation, the detailed characterization of anatomical variations provided in this study may assist radiologists in recognizing typical variation-related patterns and reducing potential diagnostic pitfalls, particularly when clinical and laboratory data are limited.

Bone marrow edema and structural changes have been identified in accessory SIJ and dysmorphic alterations, exclusively within the specified anatomical localization. These changes are associated with structural modifications in no more than three consecutive sections. These features may serve as valuable criteria for differentiation in clinical practice.

In our study of the accessory SIJ, the most prevalent variation was identified between the first and second sacral foramina, specifically in the middle third of the posterior region. This variation was predominantly unilateral and consistent with existing literature, was most accurately characterized on axial images [6]. Structural signal intensity alterations were observed, primarily manifesting as edema and notably sclerosis. Mechanical changes, such as subchondral sclerosis, subchondral cysts, and osteophytes, have been documented in studies utilizing CT, while MRI frequently reveals sclerotic, fatty, and edematous changes on the articular surfaces. It has been noted that these findings should not be misconstrued as sacroiliitis, particularly on coronal images, thereby underscoring the importance of evaluating axial images [6].

In our investigation of the iliosacral complex and sacral defect, these variations were identified as the second and third most frequently observed, respectively. They were located in the superior posterior third of the SIJ, specifically within the ligamentous portion, and were observed bilaterally. It has been documented that the increased vascular structures present in the transitional zone, where these variations are most commonly found, may extend along the bone surface at the site of the variation. These vascular structures can resemble enthesitis on coronal T2W images. Consequently, it is crucial to recognize the tubular appearance of these vascular structures on axial sections [6]. In our study, vascular structures accompanying the variation, which could be mistaken for enthesitis, were present in 42.1% of patients with the iliosacral complex and in 52.3% of patients with a sacral defect. When evaluating these variations, it is essential to assess axial images.

The prevalence of bipartite iliac bone plate was determined to be 6.8%, consistent with existing literature. This anatomical variation was most commonly identified between the first and second sacral foramina in the middle third of the posterior region on axial images and frequently presented bilaterally. In a study conducted by Puhakka et al. [15], which compared MRI and histological findings, a bone canal was described at the location corresponding to the bipartite region in the iliac bone. In certain patients, this bone canal exhibited signal characteristics indicative of a cystic structure, whereas in others, it was identified as a localized area of contrast enhancement on MRI. It was emphasized that this bone canal should not be erroneously interpreted as a signal increase; thus, recognizing this variation is crucial. In our study, a canal-like formation was observed in more than half (58.3%) of the patients with the bipartite complex variation.

The dysmorphic appearance observed in the cartilaginous portion of the joint, characterized by a single or bilateral elongated posterior joint with a convex sacral surface boundary protruding toward the adjacent iliac bone in the SIJ, was initially described by Rafei et al. [6]. This feature is located in the distal third of the joint, below the level of the second sacral foramen, and is more effectively visualized on axial sections. Given that dysmorphic changes are predominantly observed in the cartilaginous part of the joint, akin to sacroiliitis, and may be associated with sclerotic, fatty, and edematous changes in coronal images, it is crucial to recognize these findings and differentiate them from sacroiliitis [6]. In our study, dysmorphic changes were most frequently identified in the anterior inferior one-third section of the joint and were predominantly unilateral. They were most effectively detected in axial images. In 44.4% of the patients, edema accompanied the variation, while 55.6% exhibited structural signal intensity changes such as sclerosis and subchondral cysts.

The significantly higher incidence of anatomical variations in women, along with their increased prevalence with advancing age, aligns with existing literature [5,6,7,9,16]. Nevertheless, this age-related increase prompts the inquiry as to whether all detected findings genuinely represent congenital anatomical variations. It is possible that some features instead reflect age-related morphological remodeling or degenerative changes of the SIJ. A study examining pediatric SIJ morphology reported the absence of accessory SIJ, while demonstrating progressive iliac curvature with age in the region where accessory SIJ typically develops [17]. These findings suggest that certain SIJ configurations may evolve over time and may represent a combination of developmental and acquired changes. Furthermore, the higher prevalence observed in women may indicate currently under-recognized normal anatomical characteristics, which warrants further investigation [7].

This study is subject to several limitations. A significant limitation is the absence of comprehensive clinical and laboratory data. Parameters such as HLA-B27 status and inflammatory markers (CRP, ESR), which are pertinent to the diagnosis and classification of axial spondyloarthritis, were not consistently available due to the retrospective nature of the study. Additionally, patient classification was based on the referring clinician’s diagnosis rather than standardized classification criteria. Furthermore, the study population comprised only symptomatic individuals referred for SIJ MRI, which limits the generalizability of the findings to asymptomatic populations. Lastly, MRI evaluations were conducted by consensus, and interobserver agreement was not assessed. Despite these limitations, the primary objective of the study was to assess anatomical variations of the sacroiliac joint using MRI, an aim that is less reliant on laboratory data. Nevertheless, future prospective studies that incorporate clinical, laboratory, and imaging findings would offer a more comprehensive evaluation.

Consequently, variations in the SIJ are more prevalent, particularly among women. Given that these variations—primarily accessory SIJ and dysmorphic changes—can exhibit certain edematous and structural signal intensity alterations that may resemble sacroiliitis, it is crucial to recognize these variations. It is recommended that axial and coronal images be evaluated concurrently, and caution should be exercised when interpreting SIJ MR images.

## Figures and Tables

**Table 1:** Frequency of anatomical variations of SIJ *.

SIJ * Variation Type (*n* = 354)	*n*	%
Accessory SIJ *	128	36.2
Iliosacral complex	114	32.2
Semicircular defect	44	12.4
Crescent ilium	26	7.3
Bipartite iliac bone plate	24	6.8
Dysmorphic changes	18	5.1
Persistent ossification center	0	0
Isolated synostosis	0	0

* SIJ: Sacroiliac joint.

**Table 2:** Anatomical variations of SIJ *: prevalence, location, and associated bone changes.

SIJ * Variation Type	Frequency *n* (%)	Bilateral *n* (%)	Location Relative to Sacral Foramen	Anatomical Localization	MRI Findings		
					Edema *n* (%)	Structural Changes *n* (%)	Prominent Vascular Structures *n* (%)
Accessory SIJ *	128 (36.2)	49 (38.3)	Between 1st and 2nd foramina	Middle 1/3 posterior	6 (4.7)	60 (46.9) Sclerosis *n*: 50 (83.4) Fatty replacement *n*: 6 (10) Joint space narrowing *n*: 2 (3.3) Ankylosis *n*: 2 (3.3)	0
Iliosacral complex	114 (32.2)	74 (64.9)	1st foramen	Superior 1/3 posterior	0	7 (6.1) Sclerosis *n*: 7 (100)	48 (42.1)
Semicircular defect	44 (12.4)	36 (81.8)	Above 1st foramen	Superior 1/3 posterior	0	0	23 (52.3)
Crescent ilium	26 (7.3)	24 (92.3)	Between 1st and 2nd foramina	Middle 1/3 posterior	0	0	0
Bipartite iliac bone plate	24 (6.8)	19 (79.2)	Between 1st and 2nd foramina	Middle 1/3 posterior	0	0	0
Dysmorphic changes	18 (5.1)	1 (5.6)	Below 2nd foramen	Inferior 1/3 anterior	8 (44.4)	10 (55.6) Sclerosis n: 8 (80) Subchondral cyst n: 2 (20)	0

* SIJ: Sacroiliac joint.

## Data Availability

The data presented in this study are available on request from the corresponding author. The data are not publicly available due to ethical reasons.

## References

[1] Lambert R.G., Bakker P.A., van der Heijde D., Weber U., Rudwaleit M., Hermann K.G., Sieper J., Baraliakos X., Bennett A., Braun J. (2016). Defining active sacroiliitis on MRI for classification of axial spondyloarthritis: Update by the ASAS MRI working group. Ann. Rheum. Dis..

[2] Prassopoulos P.K., Faflia C.P., Voloudaki A.E., Gourtsoyiannis N.C. (1999). Sacroiliac joints: Anatomical variants on CT. J. Comput. Assist. Tomogr..

[3] Cihan Ö.F., Karabulut M., Kılınçoğlu V., Yavuz N. (2021). The variations and degenerative changes of sacroiliac joints in asymptomatic adults. Folia Morphol..

[4] Demir M., Mavi A., Gümüsburun E., Bayram M., Gürsoy S., Nishio H. (2007). Anatomical variations with joint space measurements on CT. Kobe J. Med. Sci..

[5] Teran-Garza R., Verdines-Perez A.M., Tamez-Garza C., Pinales-Razo R., Vilchez-Cavazos J.F., Gutierrez-de la O.J., Quiroga-Garza A., Elizondo-Omaña R.E., Guzman-Lopez S. (2021). Anatomical variations of the sacro-iliac joint: A computed tomography study. Surg. Radiol. Anat..

[6] El Rafei M., Badr S., Lefebvre G., Machuron F., Capon B., Flipo R.M., Cotten A. (2018). Sacroiliac joints: Anatomical variations on MR images. Eur. Radiol..

[7] Kiil R.M., Jurik A.G., Zejden A. (2022). Anatomical variation at the sacroiliac joints in young adults: Estimated prevalence by CT and concomitant diagnostics by MRI. Skeletal Radiol..

[8] Giraudo C., Magnaldi S., Weber M., Puchner A., Platzgummer H., Kainberger F., Schueller-Weidekamm C. (2016). Optimizing the MRI protocol of the sacroiliac joints in Spondyloarthritis: Which para-axial sequence should be used?. Eur. Radiol..

[9] Tok Umay S., Korkmaz M. (2020). Frequency of anatomical variation of the sacroiliac joint in asymptomatic young adults and its relationship with sacroiliac joint degeneration. Clin. Anat..

[10] Weber U., Jurik A.G., Lambert R.G.W., Maksymowych W.P. (2021). Imaging in Axial Spondyloarthritis: What is Relevant for Diagnosis in Daily Practice?. Curr. Rheumatol. Rep..

[11] Weber U., Jurik A.G., Zejden A., Larsen E., Jørgensen S.H., Rufibach K., Schioldan C., Schmidt-Olsen S. (2018). Frequency and Anatomic Distribution of Magnetic Resonance Imaging Features in the Sacroiliac Joints of Young Athletes: Exploring “Background Noise” Toward a Data-Driven Definition of Sacroiliitis in Early Spondyloarthritis. Arthritis Rheumatol..

[12] Badr S., Jacques T., Lefebvre G., Boulil Y., Abou Diwan R., Cotten A. (2021). Main Diagnostic Pitfalls in Reading the Sacroiliac Joints on MRI. Diagnostics.

[13] de Winter J., de Hooge M., van de Sande M., de Jong H., van Hoeven L., de Koning A., Berg I.J., Ramonda R., Baeten D., van der Heijde D. (2018). Magnetic Resonance Imaging of the Sacroiliac Joints Indicating Sacroiliitis According to the Assessment of SpondyloArthritis international Society Definition in Healthy Individuals, Runners, and Women With Postpartum Back Pain. Arthritis Rheumatol..

[14] Caetano A.P., Mascarenhas V.V., Machado P.M. (2021). Axial Spondyloarthritis: Mimics and Pitfalls of Imaging Assessment. Front. Med..

[15] Puhakka K.B., Melsen F., Jurik A.G., Boel L.W., Vesterby A., Egund N. (2004). MR imaging of the normal sacroiliac joint with correlation to histology. Skeletal Radiol..

[16] Ziegeler K., Hermann K.G.A., Diekhoff T. (2021). Anatomical Joint Form Variation in Sacroiliac Joint Disease: Current Concepts and New Perspectives. Curr. Rheumatol. Rep..

[17] Rixey A., Murthy N., Amrami K., Frick M., McKenzie G. (2021). The pediatric accessory sacroiliac joint: Does it exist?. Skeletal Radiol..

